# *XRCC1* R194W and R399Q Polymorphisms and Colorectal Cancer Risk in a Northeastern Mexican Population

**DOI:** 10.1155/2023/5565646

**Published:** 2023-10-04

**Authors:** Juan Pablo Meza-Espinoza, Valeria Peralta-Leal, Jorge Durán-González, Nelly Macías-Gómez, Anabel Bocanegra-Alonso, Evelia Leal-Ugarte

**Affiliations:** ^1^Facultad de Medicina e Ingeniería en Sistemas Computacionales de Matamoros, Universidad Autónoma de Tamaulipas, Sendero Nacional km 3, CP 87349, Col. San José, Matamoros, Tamaulipas, Mexico; ^2^Centro Universitario del Sur, Universidad de Guadalajara, Av. Enrique Arreola Silva # 883, Col. Centro, Ciudad Guzmán, Jalisco, Mexico; ^3^Unidad Académica Multidisciplinaria Reynosa-Aztlán, Universidad Autónoma de Tamaulipas, Calle 16 y Lago de Chapala, Col. Aztlán, Reynosa, Tamaulipas, Mexico

## Abstract

Colorectal cancer (CRC) is one of the most common cancers worldwide. Its etiopathogenesis is complex, mainly influenced by genetic instability caused by the accumulation of mutations. The *XRCC1* gene, which is involved in DNA repair, has been associated with CRC through the R194W (C194T) and R399Q (G399A) polymorphisms, but the results are inconsistent. Here, we analyzed the association of these polymorphisms with sporadic CRC in a northeastern Mexican population, including 155 male CRC patients and 155 male controls. Genotyping was performed using the RFLP method. An association with CRC was found for the 399A allele (G vs A; OR = 1.48 (1.03–2.13), *P*=0.034) and for the 399AA genotype in a codominant model (AA vs GG; OR = 3.11 (1.06–9.10), *P*=0.031). In contrast, there were no significant differences between CRC patients and controls for the C194T polymorphism (C vs T; OR = 0.82 (0.52–1.31), *P*=0.41). These results are consistent with many similar studies, but further research is needed to verify whether the *XRCC1* R194W and R399Q polymorphisms play a role in CRC etiology. The functional significance of these polymorphisms is unclear, but some studies suggest that they influence DNA repair capacity and, thus, cancer risk.

## 1. Introduction

Colorectal cancer (CRC) is one of the most common cancers worldwide, being the third most diagnosed cancer in men (10.6%), after lung cancer (14.3%) and prostate cancer (14.1%), and the second most diagnosed cancer in women (9.4%), after breast cancer (24.5%) [1]. In 2020, there were 1,930,000 CRC cases and 940,000 CRC-associated deaths, representing 10% and 9.4% of all cancers, respectively [2, 3]. CRC is classified according to its etiology as sporadic, caused by somatic mutations, which represents nearly 70% of the cases, familial (predisposition to CRC), accounting for 10–30%, and hereditary (with Mendelian inheritance), explaining around 5–7% of the cases. Its etiopathogenesis is complex and influenced by the genetic background, mainly by chromosomal and microsatellite instability, abnormal DNA methylation, and DNA repair defects [4].

High penetrance mutations, such as those in mismatch repair genes and APC gene, comprise about 5% of CRC cases, and their role in CRC pathogenesis is well established. In contrast, low-penetrance variants represent the remaining genetic factors and are poorly understood [5]. In this line, genetic polymorphisms in DNA repair genes, such as XRCC1, may contribute to differences in DNA repair capacity and thus increase susceptibility to CRC [6]. The XRCC1 gene encodes a scaffold protein that interacts with several enzymes, such as polyadenosine diphosphate (ADP)-ribose polymerase (PARP), DNA ligase III, and DNA polymerase *β* (poly*β*) to facilitate DNA single-strand breaks repair and base excision repair (BER), and thus contribute to DNA maintenance [7]. The most studied XRCC1 polymorphisms are R194W (rs1799782, C26304T, and C194T) and R399Q (rs25487, G2815A, and G399A); the former is located between the poly*β* and PARP-binding domains, while the latter is in the carboxyl-terminal side of the PARP-interacting domain [8]. These variants have been associated with CRC, but the results are inconsistent [8–35].

This study aimed to investigate whether the *XRCC1* R194W and R399Q polymorphisms are associated with CRC in a population from northeastern México.

## 2. Materials and Methods

### 2.1. Subjects

The study included 155 male patients aged 47–79 years (mean 59.7 years) with histopathologically confirmed CRC who were enrolled at the Oncology Service of the Dr. José María Cantú Hospital in Reynosa Tamaulipas, México. Some women diagnosed with CRC were excluded from the analysis due to the small sample size (less than 20). In addition, 155 cancer-free men over the age of 50 with no history of CRC or other cancers who were seen at the same hospital for other reasons were consecutively recruited as a control group. The mean age of this group was 58.4 years (50–73 years). This research was conducted according to the guidelines of the Helsinki Declaration and approved by the Ethics Committee of the Faculty of Medicine of the Universidad Autónoma de Tamaulipas, Campus Matamoros. In addition, written informed consent was obtained from the patients prior to enrollment.

### 2.2. Genotyping of the *XRCC1* C194T and G399A Polymorphisms

Genotyping was performed using the RFLP method from the DNA extracted from peripheral blood, as previously reported by Meza-Espinoza et al. The description of the methodology partially reproduces their wording [36]. Briefly, the primers used were 5′-GCCCCGTCCCAGGTA-3′ and 5′-AGCCCCAAGACCCTTTCACT-3′ for the C194T polymorphism and 5′-TTGTGCTTTCTCTGTGTCCA-3′ and 5′-TCCTCCAGCCTTTTCTGATA-3′ for the G399A variant. The PCR amplification conditions for both polymorphisms consisted of an initial denaturation at 94°C for 4 min and 35 cycles at 94°C for 30 s, 60°C for 30 s, and 72°C for 30 s, followed by a final elongation at 72°C for 4 min. The PCR products were 491 base pairs (bp) for C194T and 615 bp for G399A. Digestion was performed with the *Hpa II* enzyme for both polymorphisms. For C194T polymorphism, cleavage yielded fragments of 292 bp, 178 bp, and 21 bp for the C allele and 313 bp and 178 bp for the T allele (Supplementary Figure 1), whereas for G399A polymorphism, cleavage rendered fragments of 377 bp and 238 bp for the G allele and 615 bp (uncut) for the A allele (Supplementary Figure 2).

### 2.3. Statistical Analysis

Allele and genotype frequencies were recorded, and Hardy–Weinberg equilibrium was assessed by the chi-squared test using the control group. Pearson's chi-squared test was used to compare allele and genotype frequencies between both groups, and the association with CRC was estimated by odds ratio and 95% confidence interval (SPSS 25.0). A *P* < 0.05 was considered significant.

## 3. Results

The genotype and allele frequencies of *XRCC1* C194T and G399A polymorphisms in CRC patients and controls are shown in Table 1. The C194T genotype frequencies were 70.5%, 27.4%, and 2.1% in controls and 73.3%, 26.7%, and 0% in patients for CC, CT, and TT, respectively; the 194T allele frequency was 15.8% in controls and 13.4% in patients. For G399A, the genotypes were 59.4%, 37.4%, and 3.2% in controls and 49.7%, 41.9%, and 8.4% in patients for GG, GA, and AA, respectively, and the 399A allele frequency was 21.9% in controls and 29.4% in patients. Both polymorphisms were consistent with Hardy–Weinberg equilibrium in the controls (C194T: *χ*^2^ = 0.15, *P*=0.70; G399A: *χ*^2^ = 1.33, *P*=0.25). As shown in Table 1, an association with CRC was found for the 399A allele (OR = 1.48 (1.03–2.13), *P*=0.034) and the AA genotype in a codominant model (OR = 3.11 (1.06–9.10), *P*=0.031). In contrast, there were no significant differences in the distribution of C194T polymorphism between CRC patients and controls (OR = 0.82 (0.52–1.31), *P*=0.41).

## 4. Discussion

Our results show that subjects carrying the 399A allele have a significantly increased risk of developing CRC. This finding is consistent with observations from studies conducted in other populations, namely, Korean [9], Polish [10, 11], Han Chinese [12, 13], Japanese [14], Romanian [15], Thai [16], and Iranian [17, 18] (Table 2). Similarly, the AA genotype was associated with CRC under a codominant model in these races: Han Chinese (OR = 2.28 (1.52–3.44), *P* < 0.0001) [12] and (OR = 1.93 (1.05–3.54), *P*=0.03) [13], Japanese (OR = 1.61 (1.05–2.48), *P*=0.028) [14], Romanian (OR = 3.49 (1.55–8.02), *P*=0.001) [15], Thai (OR = 4.95 (1.99–12.30), *P*=0.0005) [16], and Iranian (OR = 5.30 (1.90–14.20), *P*=0.001) [17]. The A allele also showed an increased CRC risk under a dominant model in Korean (OR = 1.61 (1.09–2.39), *P*=0.017) [9] and Iranian (OR = 1.78 (1.16–2.74), *P*=0.009) [18]. Even the GA genotype was associated with CRC under a codominant model in Polish (OR = 2.73 (1.31–5.68), *P*=0.006) [10] and (OR = 2.48 (1.75–3.53), *P* < 0.0001) [11], Han Chinese (OR = 1.46 (1.06–2.01), *P*=0.02) [13], and Romanian (OR = 1.75 (1.09–2.82), *P*=0.017) [15]. However, similar findings have not been replicated in many other studies, especially in Taiwanese [19], Norwegian [20], Spanish [21], Polish [22, 23], Singaporean [24], Italian [25], Czech [26], American [27, 28], Mexican [29], Indian [30, 31], Northeast Chinese [32], Swedish [33], and Malaysian [34] (Table 2).

Regarding the R194W polymorphism, like us, most studies showed no association with CRC, namely, Han Chinese [13], Japanese [14], Norwegian [20], Polish [22], Italian [25], Mexican [29], and Malaysian [34]. However, some Asian studies reported a risk of CRC in carriers of the 194T allele, specifically in Han Chinese (OR = 1.30 (1.04–1.64), *P*=0.023) [8], Northeast Chinese (OR = 1.29 (1.07–1.55), *P*=0.007) [32], Korean (OR = 2.87 (2.01–4.11), *P* < 0.0001) [9], and Iranian (OR = 4.95, (2.11–11.6), *P* < 0.001) [35] (Table 3).

The discordance observed between studies for both polymorphisms is likely due to multiple factors, but perhaps racial and genetic differences, cancer histology, and inclusion criteria are the most relevant. Even this research differs from another study we have previously reported, in which no association of the R194W and R399Q polymorphisms with CRC was found in a group of patients from western México [29]. Since both studies analyzed Mexican patients, these contradictory results may be explained by the genetic background between both regions, as genetic differences between Mexican geographic areas have been demonstrated [37]. Different dietary and lifestyle habits could also be involved.

Although CRC is complex and multifactorial, oxidative stress influenced by oxidizing agents plays a role in its etiopathogenesis [11]. One of the DNA damage principal agents is known to be reactive oxygen species [38]. This damage is mainly caused by the formation of 8-oxoguanine (8-oxoG), which can cause mispairing with adenine, resulting in guanine-to-thymine and cytosine-to-adenine changes [39]. Accumulation of DNA damage due to misrepair or incomplete repair can lead to mutagenesis and subsequent transformation [40]. In this regard, an increase in oxidatively damaged DNA by 8-oxoG has been reported in leukocytes from CRC patients [41]. The removal of 8-oxoG from DNA is accomplished by BER, mainly through 8-oxo-guanine glycosylase (OGG1) activity [42], which interacts with other proteins, such as XRCC1, to maintain genomic stability. The interaction of XRCC1 with OGG1 leads to a 2- to 3-fold stimulation of the DNA glycosylase activity of this enzyme, which accelerates the overall repair process of oxidized purines and single-strand breaks [43]. The R399Q polymorphism has been shown to increase cancer risk in association with lower 8-oxoG cleavage activity and, consequently, increased levels of 8-oxoG [11, 44].

The functional significance of these polymorphisms was evaluated in individuals exposed to mutagenic agents. Regarding R399Q, the A allele (399Q) was found to contribute to ionizing radiation hypersensitivity in subjects exposed to *γ*-rays [45]; this was also associated with an increase in chromosomal deletions in individuals exposed to X-rays [46]; in addition, among subjects exposed to bleomycin and benzo[a]pyrene diol epoxide, those with the AA genotype had higher levels of chromosomal breaks than those with other genotypes [47]. In contrast, wild-type CC homozygotes for the R194W polymorphism had increased levels of chromosomal breaks [47], and subjects carrying the T allele had a reduced risk of chronic benzene poisoning [48]. It is known that benzene, through its metabolites, can induce genotoxicity and, consequently, malignancy through oxidative stress [49]. These studies demonstrate the importance of these genetic variants in the ability of *XRCC1* in DNA repair.

## 5. Conclusion

This study found that the *XRCC1* R399Q polymorphism, but not the R194W, is associated with CRC susceptibility in a population from northeastern México. However, further validation of our findings in larger samples is needed.

## Figures and Tables

**Table 1:** Analysis of the *XRCC1* C194T and G399A polymorphisms in CRC patients and controls from northeastern México.

Genotype/alleles C194T	Controls, *n* = 146	Patients, *n* = 146	OR (95% CI)	^a^ *P*
CC	103	107	1.0 (reference)	
CT	40	39	0.94 (0.56–1.57)	0.81^b^
TT	3	0	0.14 (0.007–2.70)	0.19^b^
CT + TT	43	39	0.87 (0.52–1.46)	0.60^c^
C	246	253	1.0 (reference)	
T	46	39	0.82 (0.52–1.31)	0.41

G399A	*n* = 155	*n* = 155		^a^ *P*

GG	92	77	1.0 (reference)	
GA	58	65	1.34 (0.84–2.13)	0.22^b^
AA	5	13	**3.11 (1.06–9.10)**	**0.031** ^**b**^
GA + AA	63	78	1.48 (0.94–2.32)	0.087^c^
G	242	219	1.0 (reference)	
A	68	91	**1.48 (1.03–2.13)**	**0.034**

*n*: sample size; OR: odds ratio; CI: confidence interval; *P*: *P* value. ^a^Pearson's chi-square test. ^b^Codominant model. ^c^Dominant model. Bold indicates that the A allele and the AA genotype were associated with colorectal cancer.

**Table 2:** Analysis of the *XRCC1* G399A polymorphism in populations worldwide.

^a^Country	Controls/cases	OR (95% CI)	^b^ *P* value	Reference
Korea	209/209	**1.39 (1.00–1.93)**	**0.047**	[9]
Poland	153/113	**1.51 (1.07–2.15)**	**0.02**	[10]
Poland	310/318	**1.35 (1.08–1.70)**	**0.01**	[11]
China	970/485	**1.42 (1.20–1.69)**	**0.001**	[12]
China	350/320	**1.41 (1.10–1.79)**	**0.006**	[13]
Japan	776/685	**1.19 (1.01–1.41)**	**0.04**	[14]
Romania	162/150	**2.37 (1.69–3.32)**	**0.0001**	[15]
Thailand	230/230	**1.60 (1.20–2.13)**	**0.001**	[16]
Iran	150/150	**1.54 (1.10–2.10)**	**0.01**	[17]
Iran	160/180	**1.50 (1.07–2.10)**	**0.018**	[18]
Taiwan	729/718	0.89 (0.76–1.06)	0.19	[19]
Norway	399/157	0.92 (0.71–1.21)	0.56	[20]
Spain	322/355	0.96 (0.77–1.21)	0.74	[21]
Poland	100/100	0.84 (0.56–1.26)	0.41	[22]
Poland	100/133	1.08 (0.74–1.58)	0.69	[23]
Singapore	1120/294	0.87 (0.71–1.08)	0.21	[24]
Italy	121/109	1.10 (0.74–1.63)	0.64	[25]
Czech Republic	530/532	0.94 (0.79–1.12)	0.48	[26]
USA	1950/1582	0.95 (0.86–1.05)	0.33	[27]
USA	360/305	1.00 (0.80–1.25)	1.00	[28]
México	120/103	1.21 (0.80–1.83)	0.36	[29]
India	146/120	0.77 (0.55–1.10)	0.15	[30]
India	150/130	0.89 (0.63–1.26)	0.51	[31]
China	630/451	0.99 (0.82–1.19)	0.92	[32]
Sweden	558/452	1.12 (0.94–1.35)	0.21	[33]
Malaysia	212/130	1.04 (0.74–1.46)	0.81	[34]

^a^Only case-control studies with at least 100 patients and 100 controls reporting genotype frequencies were included. Meta-analyses were excluded. ^b^Comparisons were made using Pearson's chi-square test with the G allele as the reference. Bold indicates a significance for the A allele with colorectal cancer.

**Table 3:** Analysis of the *XRCC1* C194T polymorphism in several countries.

^a^Country	Controls/cases	OR (95% CI)	^b^ *P* value	Reference
China	438/438	**1.30 (1.04–1.64)**	**0.023**	[8]
Korea	168/209	**2.87 (2.01–4.11)**	**0.0001**	[9]
China	628/451	**1.29 (1.07–1.55)**	**0.007**	[32]
Iran	140/291	**4.95 (2.11–11.6)**	**0.001**	[35]
China	350/320	0.86 (0.68–1.08)	0.19	[13]
Japan	776/685	0.99 (0.85–1.16)	1.0	[14]
Norway	399/156	1.24 (0.74–2.08)	0.41	[20]
Spain	322/360	0.89 (0.58–1.35)	0.58	[21]
Poland	100/100	1.11 (0.46–2.67)	0.82	[22]
Poland	100/133	1.76 (0.71–4.37)	0.21	[23]
Singapore	1162/305	0.93 (0.76–1.24)	0.48	[24]
Italy	121/109	1.26 (0.64–2.50)	0.50	[25]
USA	1950/1582	1.19 (0.97–1.45)	0.09	[27]
USA	360/305	0.86 (0.58–1.29)	0.46	[28]
México	120/107	0.60 (0.34–1.06)	0.07	[29]
Malaysia	212/130	0.96 (0.68–1.35)	0.82	[34]

^a^Only case-control studies with at least 100 patients and 100 controls reporting genotype frequencies were included. Meta-analyses were excluded. ^b^Comparisons were made using Pearson's chi-square test with the C allele as the reference. Bold values indicate a significance for the T allele with colorectal cancer.

## Data Availability

The datasets generated and analyzed in this study are available from the corresponding author upon request.

## Supplementary Materials

Supplementary Materials Supplementary Figure 1: photograph showing results of the XRCC1 C194T polymorphism. Lanes 1 and 4–8: wild homozygotes (CC). Lanes 2 and 3: heterozygotes (CT). Lane 10: polymorphic homozygote (TT). Lane 9: 100 bp marker. Supplementary Figure 2: photograph showing results of the *XRCC1* G399A polymorphism. Lanes 1, 3, 4, and 7–11: heterozygotes (GA). Lanes 2 and 12: wild homozygotes (GG). Lane 6: polymorphic homozygote (AA). Lane 5: 100 bp marker.
