# Supplementary material for: XRCC1 R194W and R399Q Polymorphisms and Colorectal Cancer Risk in a Northeastern Mexican Population
Source: Genet Res (Camb). 2023 Oct 4;2023:5565646. doi: 10.1155/2023/5565646 (PMC10567464; doi:10.1155/2023/5565646)
Supplement: Supplementary Materials — Supplementary Figure 1: photograph showing results of the XRCC1 C194T polymorphism. Lanes 1 and 4–8: wild homozygotes (CC). Lanes 2 and 3: heterozygotes (CT). Lane 10: polymorphic homozygote (TT). Lane 9: 100 bp marker. Supplementary Figure 2: photograph showing results of the XRCC1 G399A polymorphism. Lanes 1, 3, 4, and 7–11: heterozygotes (GA). Lanes 2 and 12: wild homozygotes (GG). Lane 6: polymorphic homozygote (AA). Lane 5: 100 bp marker. [file 5565646.f1.zip › Figure Supplementary 2.docx]

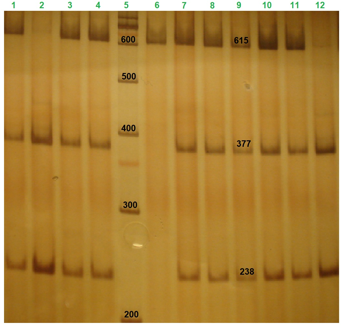


**Figure supplementary 2.** Photograph showing results of the *XRCC1* G399A polymorphism. Lanes 1, 3, 4, 7-11: Heterozygotes (GA). Lanes 2 and 12: Wild homozygotes (GG). Lane 6: Polymorphic homozygote (AA). Lane 5: 100-bp marker.
